# Evaluation of the Effectiveness of a Novel Wireless Energy-Transmitting Implantable Diaphragm Pacemaker in Anesthetized Pigs

**DOI:** 10.3390/bioengineering13040469

**Published:** 2026-04-16

**Authors:** Xiaoyu Gu, Wei Zhong, Zhihao Mao, Yan Shi, Yixuan Wang

**Affiliations:** 1 School of Mechanical Engineering, Jiangsu University of Science and Technology, Zhenjiang 212100, China; zhongwei@just.edu.cn; 2 School of Biology Science and Medical Engineering, Beihang University, Beijing 100191, China; 3 Department of Neurosurgery, Xintai Hospital of Traditional Chinese Medicine, Xintai 271200, China; mzhabcd567@163.com; 4 School of Automation Science and Electrical Engineering, Beihang University, Beijing 100191, China; shiyan@buaa.edu.cn; 5 Engineering Practice and Innovative Center, Beihang University, Beijing 100191, China

**Keywords:** wireless energy transmission, implantable diaphragm pacemaker, thermal effect simulation, phrenic nerve stimulation, in vivo and in vitro testing

## Abstract

**Objectives:** This study aimed to demonstrate the feasibility of a novel wireless energy-transmitting implantable diaphragm pacemaker for restoring respiratory ventilation. **Methods:** The diaphragm pacing (DP) system was designed based on the principle of electromagnetic resonance coupling. The safety of device implantation was analyzed through finite-element simulations of multi-field coupling between electromagnetic heating and biological tissue. In vitro testing with coils embedded in pork demonstrated the system output characteristics. This device was used in miniature Bama pigs that underwent deep anesthesia and respiratory arrest (*N* = 8). Respiratory airflow, diaphragmatic displacement, and blood gases were used to evaluate the effectiveness of the designed DP system. **Results:** Thermal effect simulation results show that the temperature rise of the surrounding tissue does not exceed 2 °C during 1 h of transmission power (0.5–1.3 W) operation of the receiver. In vitro tests with two receivers embedded in pork showed that the DP system can effectively output stimulation waveforms over a certain transmission distance (5–35 mm). The stimulation waveform output by the receiver is consistent with the parameters set by the external controller. In phrenic nerve electrical stimulation experiments, the peak respiratory airflow and tidal volume remained stable over 50 consecutive respiratory cycles. The tidal volume (108.63 mL) and diaphragmatic displacement (0.883–2.15 cm) in a pig induced by DP demonstrate the effectiveness of respiratory ventilation. The arterial blood gas analysis results and temperature rise experiment during implantation further confirmed the effectiveness and safety of the ventilation. **Conclusions:** The implantable diaphragmatic pacemaker developed in this study exhibits good thermal safety, stable output, and effective respiratory ventilation. A control group with commercial diaphragmatic pacemakers and data from chronic implantation experiments are needed to further evaluate its effectiveness.

## 1. Introduction

Chronic respiratory failure caused by diseases such as high spinal cord injury (SCI), amyotrophic lateral sclerosis (ALS), or central hypoventilation syndrome (CHS) seriously affects patients’ quality of life and long-term prognosis [1,2]. A common physiological feature of these conditions is that the diaphragm, the main respiratory muscle, cannot respond to signals from the central nervous system, leading to loss of voluntary breathing function [3]. The current standard treatment for these patients is long-term reliance on ventilators for invasive or non-invasive mechanical ventilation. However, long-term mechanical ventilation is accompanied by a series of serious complications, such as disuse atrophy of the diaphragm, airway damage, and ventilator-associated pneumonia, which greatly reduce the quality of life of patients and increase the medical burden [4,5].

Implantable diaphragm pacing (DP), as a ventilation replacement therapy, works by electrically stimulating the phrenic nerve to induce regular diaphragmatic contractions, thereby restoring the patient’s spontaneous breathing ability [1,2]. Existing commercially available implantable diaphragmatic pacemakers have been shown to improve clinical outcomes, reduce ventilator dependence, and enhance quality of life in selected patients [1]. However, the high cost, implantation techniques, and specialized equipment maintenance required for these systems limit their widespread adoption globally [6]. Therefore, developing a novel, compact, cost-effective alternative DP is of great significance for expanding the accessibility of this therapy and optimizing its efficacy.

Diaphragmatic pacing requires a continuous high-current output to drive the muscle; hence, energy management is a challenge in its design. The encapsulation of batteries in implantable devices limits their lifespan and requires frequent surgeries to replace them [7]. Battery leakage can also pose a threat to patient safety [8]. Thence, the current mainstream solution uses a rechargeable battery in conjunction with external wireless charging [9,10]. Wireless power transfer (WPT) technology was first applied to medical devices in the 1960s, based on the principle of inductive coupling to power artificial hearts [11,12]. However, the transmission efficiency of this system is highly dependent on the alignment and distance between the transmitting and receiving coils [13]. Electromagnetic resonant-coupling wireless power transmission technology can overcome the main limitations of inductive coupling in terms of transmission distance and position misalignment [14]. Therefore, for surgeons, there is no need to strictly align the two coils axially during the procedure, thereby reducing the difficulty of receiver implantation. Nevertheless, most publications focus on optimizing coil design and transmission efficiency, as well as magnetic resonance coupling [15,16,17,18]. Research on the safety assessment and in vivo efficacy verification of this technology in implantable medical devices is relatively limited. Furthermore, the previous literature [19] focused on the system’s principles and the feasibility of its application in small animals (rabbits) using hook electrodes. Small animal experiments alone are insufficient to adequately support the efficacy validation required for rigorous preclinical evaluation of the DP system.

The incomplete synchrony of left- and right-sided diaphragm contractions and unilateral diaphragm dysfunction require a DP system with independent dual-channel stimulation capability. However, few improved DP systems have demonstrated the ability to flexibly adjust stimulation parameters [20,21]. For the vast majority of patients, an implantable diaphragmatic pacemaker is a long-term, and even lifelong, medical companion [22]. Therefore, designing a compact, integrated diaphragm pacemaker could significantly improve patient comfort.

High-frequency alternating magnetic fields can induce eddy currents in human tissues, causing the tissues to heat up. The high temperature of the implant can cause varying degrees of pain, inflammation, and tissue damage [23,24]. Therefore, analyzing the temperature changes within the implant is particularly necessary. Directly measuring the thermal effect between electromagnetic waves and the human body is subject to limitations imposed by experimental conditions and ethical constraints. Previous studies have mostly focused on the current generated by electromagnetic fields inside implants, without fully considering heat transfer [25,26]. Electromagnetic heat transfer simulation analysis can predict the temperature distribution in tissues surrounding the implant, ensuring that its temperature rise is controlled within a safe threshold. Moreover, the size of the implant directly affects the inflammatory response of biological tissues and the size of the wound.

The PCB planar coil is small in line width (<0.5 mm), thin in thickness (≤2 mm), and simple in manufacturing process. Using printed circuit board technology to fabricate the receiving coil can effectively reduce the implant’s size, improve preparation precision, and facilitate integration and miniaturization [27,28].

In this paper, a novel implantable diaphragm pacing (DP) prototype—comprising an external controller, two transmitter modules, and two receiver modules—was designed, fabricated, and evaluated. First, the stimulation generation, control, and power management units were integrated into the external controller based on a microcontroller. A wireless power supply for the implantable diaphragm pacemaker was realized using PCB spiral coils as the energy-transfer medium, leveraging the principle of electromagnetic resonant coupling. Second, during wireless power transmission, a finite-element simulation was employed to calculate tissue temperature, verifying the safety of the implanted receiving coil. In vitro experiments with pork as the test medium were conducted to validate the output characteristics of the DP system. We improved the electrodes in the DP system, replacing them with Cuff electrodes, which are more suitable for clinical applications. Finally, we hypothesized that the proposed DP system in pigs could elicit effective, rhythmic diaphragmatic contractions, without excessive heating or tissue damage. To test this hypothesis, peak airflow, tidal volume, arterial blood gas, diaphragm excursion, and temperature rise in Bama pigs were investigated during DP with various stimulation parameters.

## 2. Methods

### 2.1. Design of Implantable DP System

The implantable DP system consists of electrodes that contact the nerve, a wireless receiver implanted under the skin, a transmitter module assembly attached to the skin surface, and a battery-powered external controller. The design concept involves controlling an external controller to generate stimulation signals that meet the patient’s respiratory needs. These electrical stimulation signals are then transmitted wirelessly into the body, thereby stimulating the phrenic nerve.

**Figure 1:**
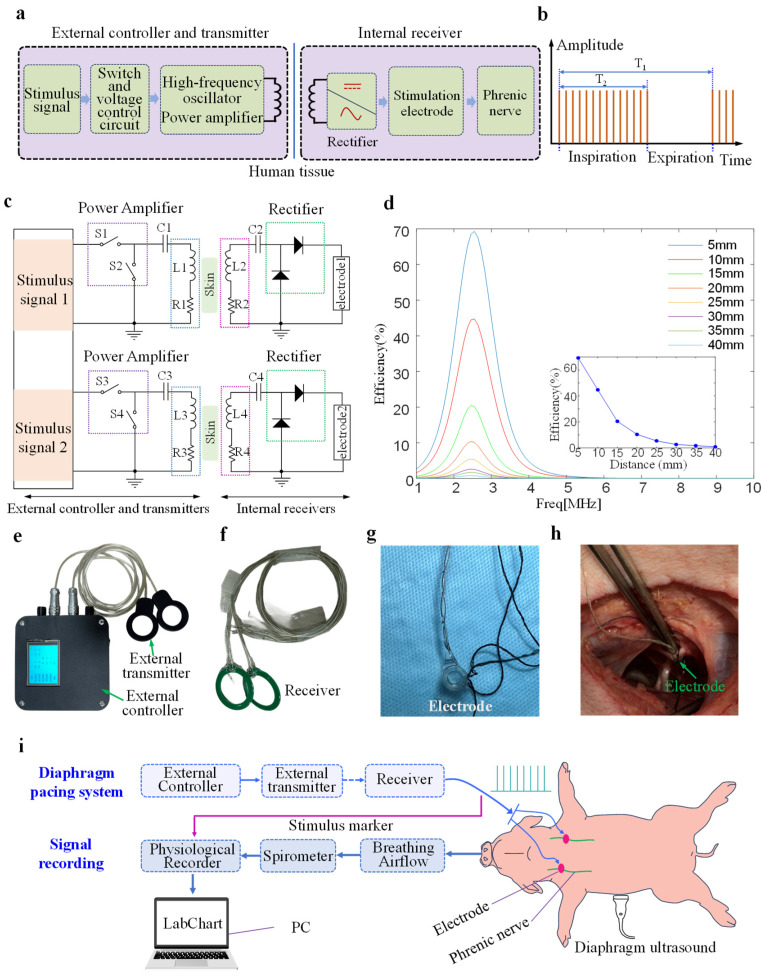
Design and experimental principle of the implantable diaphragm pacemaker. (**a**) Design and working principle of the designed diaphragm pacemaker. (**b**) Output stimulation waveform of the diaphragm pacemaker. (**c**) DP system architecture diagram. (**d**) Wireless power transfer efficiency. (**e**) External controller and external transmitter of the designed diaphragm pacemaker. (**f**) Implanted receiver. (**g**) Implantable cuff electrode. (**h**) A cuff electrode wrapped around the phrenic nerve of a pig. (**i**) Principle of signal recording during diaphragm pacing in a pig.

Figure 1a shows the principal block diagram of the designed DP system. It consists of an external part and an internal part. The design of wireless transdermal transmission for an implantable DP system is based on electromagnetic resonant coupling. The resonance between the external wireless transmitter and the internal receiver generates a changing current that is transmitted to the transmitting coil. A changing magnetic field induces a current in the receiving coil, enabling wireless power transmission between the inside and outside of the body. The external controller adjusts the stimulation parameters and controls the signal transmission. The circuit of the external transmitter consists of a switch and a voltage control circuit, a high-frequency oscillator, and a power amplifier. The rectifier circuit in the receiver inside the body rectifies the induced current generated by the receiving coil to form a square wave, which is then transmitted to the phrenic nerve. Moreover, the system’s transmitting and receiving modules operate in parallel without interference. The signals generated by the two channels can be controlled independently by the external controller.

Figure 1b illustrates the time series of the output stimulus waveform of the receiver. A respiratory cycle (T_1_) typically refers to one complete inhalation and exhalation. The output waveform during the inhalation phase (T_2_) consists of multiple identical pulsed square waves. No pulse signal is transmitted during the exhalation phase. Figure 1c shows the proposed DP system architecture. External transmitters convert direct current to alternating current through inverters and radiate it outward, while electromagnetic induction couples it to the internal receiver. Figure 1d shows the transmission efficiency of the transmitting and receiving coils in the DP system at different transmission distances, calculated using HFSS software (ANSYS Electronics Desktop 2021 R1). Increasing the distance between the transmitting and receiving coils leads to a decrease in transmission distance. At a transmission distance of 5 mm, the transmission efficiency can reach nearly 90%. Within 20 mm, the transmission efficiency is highly sensitive to changes in distance, exhibiting a steep decline. However, increasing the distance beyond 20 mm results in a more gradual change in efficiency. At transmission distances greater than 15 mm, the transmission efficiency drops below 50%.

Figure 1e is the external controller and external transmitter with the integrated circuit packaged. The external controller is equipped with an LED display that shows the stimulation parameters (amplitude, frequency, pulse width, respiratory cycle, inspiratory-expiratory ratio). Stimulation parameters of two independent channels were adjusted via two DIP switches. Figure 1f shows two receivers packaged in polydimethylsiloxane (PDMS, Dow Corning, Midlandm, MI, USA), which has good biocompatibility and sealing properties [29].

Two Cuff electrodes (Inner diameter: 1.5/2 mm, KedouBC, Suzhou, China) connected to the output terminal of receivers were implanted at the bilateral phrenic nerve sites. Figure 1g shows the structure of the electrode. The Cuff electrode is made entirely of flexible silicone material. Its wrap-around design allows for better adhesion to the nerve and insulation from surrounding tissues. This avoids external interference during stimulation. Figure 1h illustrates that the Cuff electrode was fixed around the phrenic nerve during the in vivo experiment.

### 2.2. Simulation of In Vivo Tissue Temperature

In wireless energy transmission, the heating effect of the high-frequency electromagnetic field between coils can cause thermal damage to human tissue. Finite-element analysis software (Ansys Workbench, ver. 2021 R1, Canonsburg, PA, USA) was used to analyze the thermal effect of the implanted coil of the DP. The simulation study includes a magnetic field module and a bioheat transfer module. The magnetic field module mainly studies the electromagnetic properties of the energy-transfer coil group itself and the biological tissues within its radiation range. The Bioheat Transfer Module, coupled with the physics of the Electromagnetic Module, focuses on analyzing temperature changes in biological tissues caused by electromagnetic heating.

A three-layered model structure consisting of skin, fat, and muscle was applied to simulate the human biological environment. The thickness of the skin and fat in the biological model is 3 mm, and the length and width are 54 mm. During the simulation calculation, the transmitting coil is located outside the tissue, 2 mm from the skin surface. The receiving coil is embedded in the muscle. The distance between the two coils, in vitro and in vivo, can be set to 12 mm, 18 mm, 20 mm, or 25 mm. In the simulation, the two coils are considered to be in relative facing positions, with offsets and deflections to simulate the operation of an actual wireless energy transmission system. The diameters of the transmitting coil and the receiving coil are 50 mm and 36 mm, respectively, and the corresponding numbers of turns are 7 and 6.

The Transient Thermal module was used to simulate the safety assessment of human tissue temperature, and a heat source was added through the HFSS electromagnetic physics interface. The effects of vasoconstriction, vasodilation, and sweat gland heat dissipation were ignored in the simulation [30], and the initial temperature was set to 36.2 °C. The simulation calculation time is set to 1 h, focusing only on temperature changes in human tissue. The solver used an adaptive algorithm to dynamically determine the time step to ensure the accuracy of the simulation results [23]. In addition, according to the International Organization for Standardization’s ISO 14708-1 temperature safety requirements for implantable medical devices, the temperature of tissue near an implanted device must not exceed normal human body temperature by more than 2 °C [31].

### 2.3. In Vitro Testing

To ensure the reliability of the proposed implantable diaphragm pacemaker in living pigs, in vitro testing of the system is required. Fresh minced pork placed in acrylic boxes was used as a carrier for in vitro testing. Two receivers were implanted into the pork tissue at depths ranging from 3 to 35 mm. Different implantation depths were marked on the side of the acrylic plate using a marker. Each receiver was connected to a load resistor, and the digital oscilloscope collected the electrical stimulation waveform across the load resistor. The stimulation amplitude was adjusted by a dip switch of the external controller while the system remained in operation. The remaining stimulation parameters set by the controller, such as frequency, pulse width, respiratory cycle, and inspiratory time, are consistent with those required when conducting experiments on living animals. The resistance of the human body between electrodes is approximately between 1 kΩ and 3 kΩ. Therefore, kΩ-level resistors are usually used for load testing when designing stimulators [32,33]. Amplitudes 1 kΩ, 1.5 kΩ, and 2 kΩ were selected to carry out in vitro testing of the DP system.

### 2.4. In Vivo Testing

(1) Animal models and anesthesia

Eight miniature Bama pigs, aged about 5–6 months (20 ± 2 kg), were used in an in vivo experiment of the implanted DP system. Pigs were used as experimental subjects to validate the effectiveness of the DP system and to demonstrate its potential for clinical application. Animals should be fasted for 12–24 h and deprived of water for 6–10 h before surgery. The surgical site must be cleaned and disinfected before surgery. Animals were anesthetized using intramuscular injection and air anesthesia. The pig was injected intramuscularly with Dormicum (0.01 mL/kg) before tracheal intubation. Anesthesia was maintained by tracheal intubation with isoflurane (1%~6%).

This experiment was conducted in the operating room, and the entire surgical procedure was performed under sterile conditions. On the operating table, the experimental animals were placed in a supine position. The animals’ condition was monitored throughout the surgery by a professional anesthesia technician. Electrocardiogram, blood pressure, blood oxygen saturation, and other indicators were continuously monitored to ensure stable hemodynamics and vital signs in the animals. The animal experimental protocol in this study was reviewed by the IACUC of Golden Wing (SuZhou) Medical Technology CO., Ltd. (Suzhou, China), which found it to comply with the study ethics for laboratory animals and approved its implementation (GW-IACUC-2025-054). This was not a human clinical trial, and therefore, the clinical trial number was not applicable.

(2) Surgical procedures

After general anesthesia, endotracheal intubation was performed to establish a breathing channel. The femoral artery was carefully dissected, and the distal end was ligated. The proximal end was clamped with an arterial clamp. A small oblique incision was made in the carotid artery between the arterial clamp and the ligation site for arterial cannulation.

Bilateral cervical incisions were made to expose the bilateral phrenic nerves, which were then carefully dissected from the surrounding fascia and vascular structures. Subsequently, cuff electrodes were fitted onto each isolated phrenic nerve. The input terminal of the electrode is connected to the output terminal of the receiver. After completing the dissection of the bilateral phrenic nerves and electrode implantation in the pig, blood gas measurements were performed under mild anesthesia. Then, the anesthesia concentration was increased for approximately 90 s to perform blood gas measurements under deep anesthesia.

In studying the effects of electrical stimulation parameters on diaphragmatic displacement and the effects of different transmission distances on respiratory airflow, the receivers were placed externally. To investigate the effects of stimulation parameters on respiratory airflow maintenance and the influence of receiver implantation on tissue temperature rise, two receivers were implanted subcutaneously in the abdomen.

(3) Respiratory airflow and tidal volume

The endotracheal tube was connected to the breathing flow head (MLT300L, ADInstruments, Sydney, Australia). A precision differential pressure sensor (FE141, Spirometer, ADInstruments, Sydney, Australia) connected to the flow head can collect respiratory airflow signals. The zero-crossing threshold method can be used to capture the respiratory airflow for each respiratory cycle [34]. Tidal volume can be obtained by integrating over time for each respiratory cycle.

As shown in Figure 1i, the physiological recorder (PL3516, PowerLab 16/35, ADInstruments, Sydney, Australia) records the outputs of the two DP system receivers, which verify the effectiveness of the stimulation signal delivered to the phrenic nerve. Labchart acquisition software (Lab Chart 8, ADInstruments, Sydney, Australia) can display respiratory airflow signals and stimulus marker signals. The sampling frequencies for respiratory airflow and stimulation signals were 2000 Hz and 40 kHz, respectively. Features were extracted from all signals using MATLAB software (v. R2023b, Natick, MA, USA).

(4) Ultrasound evaluation of the diaphragm

During ultrasound examination of diaphragm motion, the probe was placed in the subcostal space and pointed cranially. Diaphragmatic excursion was assessed via a subcostal approach. By placing the ultrasound probe under the costal margin or between the costal spaces, the diaphragm movement can be observed and measured in real time. Diaphragmatic excursion was defined as the vertical distance between the baseline (end of expiration) and the peak (end of inspiration during pacing) in an M-mode tracing graph. The M-mode has excellent axial and temporal resolution for diaphragmatic motion analysis [35]. In B-mode ultrasound, the liver or spleen is used as the acoustic window. Scanning is performed towards the head and back. The diaphragm appears as a hyperechoic bright line covering the surface of the liver or spleen, moving towards the probe during inhalation. All ultrasound examinations during diaphragmatic pacing are performed by an experienced ultrasound technician. Measurements were taken for three consecutive pacing cycles, and the average value was used for analysis.

(5) Phrenic nerve stimulation protocol

Various stimulation amplitudes (4 V, 5 V, 6 V, 7 V, 8 V, 9 V) were used to assess their effects on respiratory airflow, diaphragmatic displacement, and blood gases in anesthetized pigs. Different frequencies (25 Hz, 50 Hz, 100 Hz) were also used to observe the effect on the respiratory airflow waveform. In animal experiments, the remaining stimulus parameters (pulse width: 150 μs; inhalation time: 0.5 s; respiratory cycle: 1.5 s) remained unchanged.

In the experiment, to observe the effect of stimulation parameters on bilateral diaphragm displacement, two receivers were not implanted subcutaneously. Two pairs of external transmitters and receivers were placed facing each other on the operating table. Respiratory airflow and corresponding tidal volume data at various transmission distances (5/10/15/20/25/30 mm) using the same stimulation parameters (6 V, 150 μs, 50 Hz, 0.5 s, 1.5 s) were analyzed.

(6) Temperature test experiment

Temperature was monitored using a TMP119 temperature sensor (Texas Instruments, Dallas, TX, USA). During the DP experiment, the sensor was placed directly below the receiving coil and attached to the abdominal tissue of the pig. The experiment lasted for 60 min, with temperature data plotted every 5 min. The experiment mainly tested temperature changes at different stimulus amplitudes (4 V, 6 V, 9 V), while other stimulus parameters (150 μs, 50 Hz, 0.5 s, 1.5 s) remained constant.

(7) Statistical processing

The experiment was divided into a control group and experimental groups with different stimulation parameters. The control group received no electrical stimulation. Corresponding respiratory airflow signals were recorded under deep anesthesia. Each experimental group received different stimulation parameters and transmission distances. Quantitative data are expressed as mean ± standard deviation and reported with 95% confidence intervals (95% CI). Peak respiratory airflow, tidal volume, blood gas values, and implant temperature rise in the eight pigs under different testing conditions were compared using a paired-samples *t*-test. All statistical analyses were performed in MATLAB, with a significance level of α = 0.05; *p* < 0.05 was considered statistically significant.

## 3. Results

### 3.1. Temperature Safety of DP

**Figure 2:**
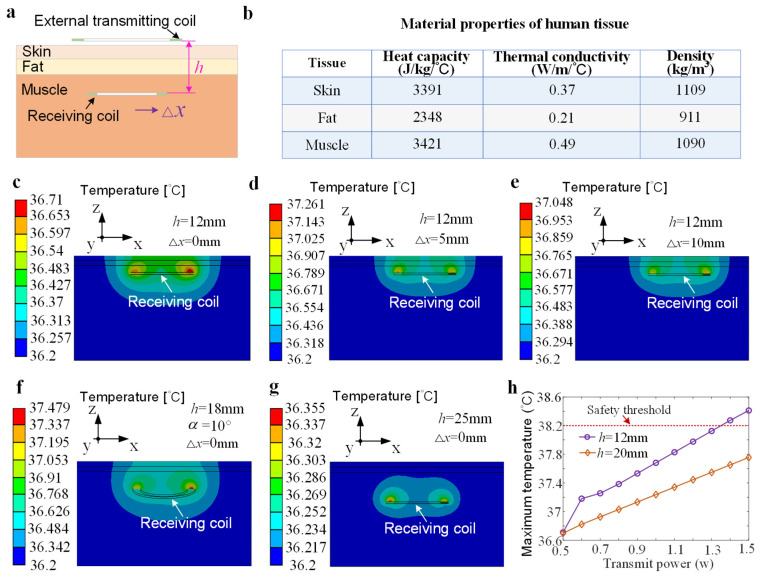
Simulation of human tissue temperature distribution during wireless energy transmission. (**a**) Two-dimensional model of coil implantation in human tissue. (**b**) Human tissue parameters at an operating frequency of 2.5 MHz. (**c**) Temperature distribution of human tissue with two coils facing each other (*h* = 12 mm). (**d**,**e**) Simulations of temperature distribution with two coils being offset (Δ*x* = 5 mm/10 mm, *h* = 12 mm). (**f**) Temperature distribution with the implanted coil being deflected 10° around the *x*-axis (*h* = 18 mm). (**g**) Temperature distribution simulation (*h* = 25 mm). (**h**) Maximum temperature of human tissue at various transmit powers (0.5–1.5 W).

Figure 2a shows the simulation model of the diaphragm pacing wireless power supply system. The transmitting and receiving coils adopt a circular spiral structure and are designed on the printed circuit board. The values of the heat capacity, thermal conductivity, and density of the human tissue at an operating frequency of 2.5 MHz are shown in Figure 2b. Figure 2c–g represent the tissue temperature distribution of the implanted coil at different positions in the body when the transmission power is 0.5 W. When the coil group is in the facing position with a corresponding transmission distance of 12 mm (Figure 2c) and 25 mm (Figure 2g), the maximum temperatures are 36.71 °C and 36.355 °C, respectively. The calculated temperature rise is 0.51 °C and 0.155 °C. The temperature rise is significantly increased when the coil group is offset compared to the aligned condition (Figure 2d,e). For the same transmission distance (*h* = 12 mm), the temperature rise corresponding to the offset distances of 5 mm and 10 mm is 1.061 °C and 0.848 °C, respectively. The relative deflection of the two coils also significantly increases the temperature rise. Figure 2f shows that the maximum temperature of human tissue is 37.479 °C, with a temperature rise of 1.279 °C. At a transmission power of 0.5 W, the temperature changes corresponding to various relative offset and deflection positions of the two coil groups were all less than 2 °C, which is lower than the temperature rise limit specified by the ISO standard. Since the transmitting coil is placed outside the body, air flow effectively reduces the temperature rise. Hence, the temperature rise is mainly concentrated in the biological tissue near the receiving coil. Figure 2h illustrates the maximum temperature values of the in vivo tissue at various transmission powers (0.5–1.5 W) when the axial transmission distance between the two coils is 12 mm and 10 mm. It can be seen that increasing transmit power leads to an increase in temperature. When the transmit power is less than 1.3 W, the temperature rise at both transmission distances is below the safety threshold. The temperature rise is closely related to the axial distance between the two coils. The increase in the axial distance reduces the temperature rise.

### 3.2. Output Characteristics of the DP System with Receivers Buried in Pork Mince

**Figure 3:**
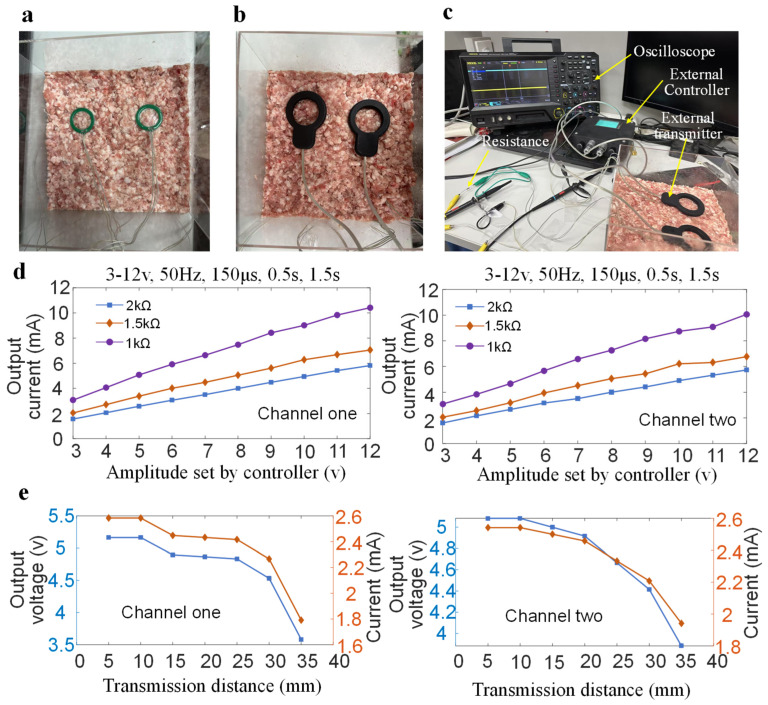
Measurement of the proposed DP system with two receivers buried in minced pork meat. (**a**) Two receivers are placed on the minced meat. (**b**) Two transmitters are placed above the receiver, which is buried in the pork. (**c**) Output test scenario of the DP system. (**d**) Output current of two receivers under different amplitude settings by the external controller. (**e**) Output voltage and current of two receivers under different transmission distances with the same stimulation parameters.

To verify the effectiveness of the system output and receiver packaging, the receiver was buried in minced pork (Figure 3a). Two external transmitters were placed directly above the two receivers (Figure 3b). Resistors of varying values were connected to the receiver outputs, and the output characteristics were analyzed using an oscilloscope (Figure 3c). The external controller sets different stimulation amplitudes (3–12 V), and the remaining stimulation parameters remain the same (frequency: 50 Hz, pulse width: 150 μs, inspiratory time: 0.5 s, respiratory cycle: 1.5 s). The oscilloscope detects the output voltage of the receiver connected to three types of resistors (2 kΩ, 1.5 kΩ, 1 kΩ). The output current was then calculated using Ohm’s law. As shown in Figure 3d, increasing the amplitude of the external controller also increases the output current. Increasing load resistance decreases output current. The output current range of the receiver connected to a load resistor of 2 kΩ is 1.574–5.824 mA. Figure 3e shows how the receiver output changes with the transmission distance under the same stimulation parameters (amplitude: 5 V; frequency: 50 Hz; pulse width: 150 μs; inspiratory time: 0.5 s; respiratory cycle: 1.5 s; load resistance: 2 kΩ). It can be seen that increasing the transmission distance decreases the output voltage and current. At a transmission distance of 5 mm, the receiver’s output voltage and current for channel 1 are 5.166 V and 2.583 mA, respectively. The output voltage and current drop to 3.583 V and 1.7915 mA with the transmission distance of 35 mm. When the transmission distance is less than 20 mm, the decrease in the receiver output voltage and current values is small. The output currents of the two receivers are 1.7915 mA and 1.9415 mA, with a transmission distance of 35 mm.

### 3.3. Respiratory Airflow, Diaphragm Excursion, and Temperature Rise Induced by the DP System

**Figure 4:**
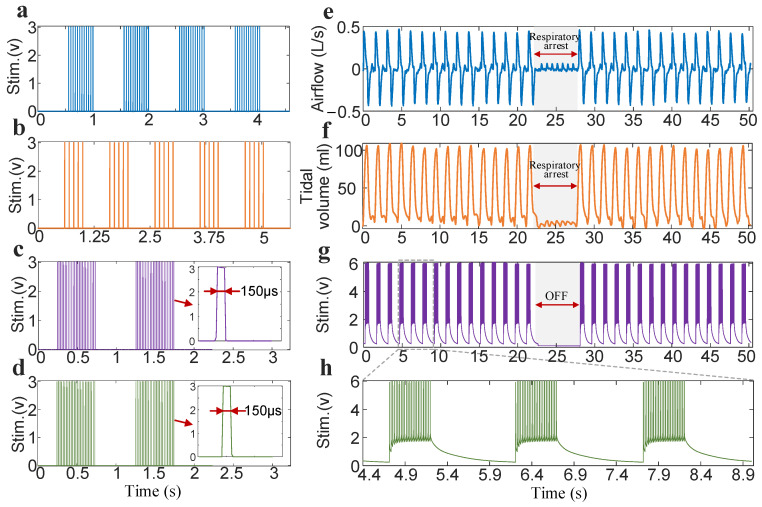
DP system’s stimulation waveform output and ability to continuously drive respiratory ventilation. (**a**) Stimulation waveform output of four consecutive respiratory cycles for one receiver at 25 Hz. (**b**) Stimulation waveform of five consecutive respiratory cycles at 10 Hz. (**c**,**d**) Synchronous output from two receivers at 50 Hz. (**e**) A representative set of continuous diaphragmatic-paced respiratory airflow. It contains 30 respiratory cycles. The gray shading indicates that the DP system is off, and no stimulation signal is sent to the phrenic nerves. (**f**) A representative set of diaphragm-paced respiratory tidal volume waveforms. (**g**) Stimulation signals sent by the receiver recorded by the physiological recorder during DP. (**h**) Local amplification of the stimulus markers.

The output terminals of the two receivers were each connected to two 2 kΩ resistors. The stimulation parameters (amplitude: 3 V; pulse width: 150 μs; inspiratory time: 0.5 s; respiratory cycle: 1 s) remained constant when the output voltage was tested with an oscilloscope. Figure 4a,b show the electrical stimulation waveforms continuously output by one of the receivers in the DP system at frequencies of 25 Hz and 10 Hz. Notably, the time-series characteristics of the stimulation waveform are basically consistent with the settings of the external controller. At a frequency of 25 Hz and an inspiratory time of 0.5 s, the number of pulses in one respiratory cycle is 12. The corresponding number of pulses at 10 Hz is 5. Figure 4c,d are the stimulation waveforms output simultaneously by two receivers at a frequency of 50 Hz within two respiratory cycles. The output waveforms of the two receivers are synchronized in the time series. A magnified view of a single pulse shows that the waveform does not exhibit obvious overshoot or undershoot.

In a respiratory segment during a subject’s DP process (Figure 4e,f), we demonstrated that the designed system can increase peak respiratory flow and tidal volume to 0.435 L/s and 108.63 mL, respectively. When diaphragmatic pacing (stimulation parameters: 6 V, 150 μs, 50 Hz, 0.5 s, 1.5 s) was briefly paused and then reactivated, it was found that it could immediately restore airflow and produce a ventilation effect. The peak respiratory airflow and tidal volume produced were essentially consistent with the effect before DP was turned off. The stimulus markers output by the DP system and collected by the physiological recorder are shown in Figure 4g. It can be observed that the designed system can stably output stimulus parameters. Figure 4h depicts a magnified view of the stimulus markers for three consecutive respiratory cycles in Figure 4g. It was found that stimulation frequency (50 Hz), respiratory time (0.5 s), and respiratory cycle (1.5 s) can be kept consistent with the settings of the external controller.

**Figure 5:**
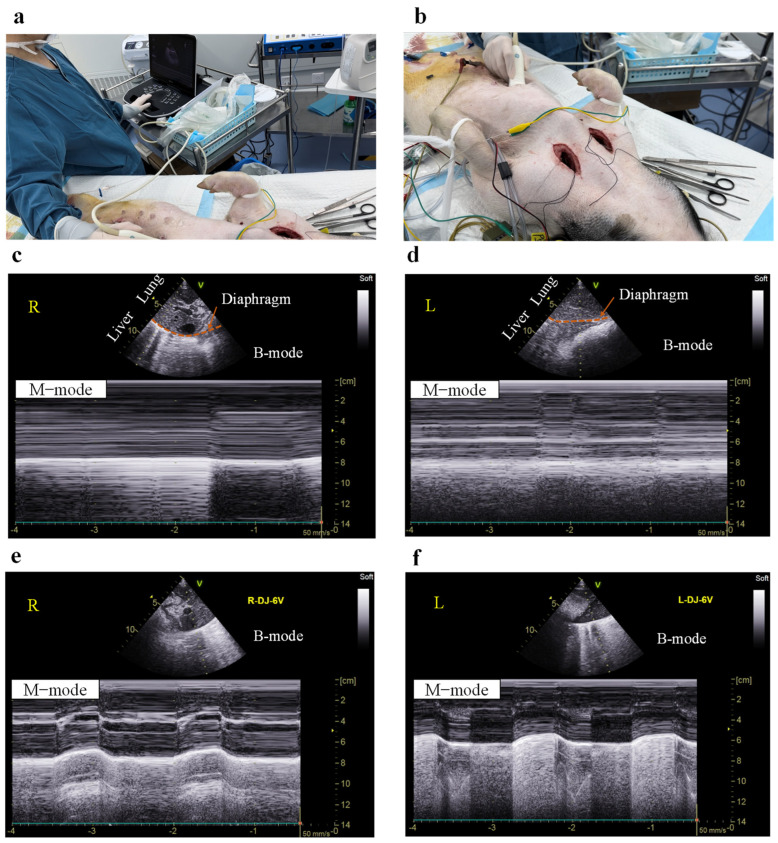
Imaging of the diaphragm and its excursion before and after DP. (**a**,**b**) Diaphragm ultrasound imaging detection scenario. (**c**,**d**) M-mode assessment of right (**c**) and left (**d**) diaphragm motion in a deeply anesthetized pig. (**e**,**f**) M-mode evaluation of right (**e**) and left (**f**) diaphragmatic excursion during DP in an anesthetized pig.

Electrical stimulation of the phrenic nerve increases the displacement of the diaphragm. Ultrasound was used to observe and quantify diaphragm displacement during DP (Figure 5). Two-dimensional (B-mode) ultrasound examination of the diaphragm can clearly provide a coronal cross-sectional image. To quantify diaphragmatic movement under bilateral phrenic nerve stimulation, we used M-mode ultrasound (Figure 5c–f). This method can display the diaphragmatic movement trajectory along a selected line within the B-mode image. Figure 5a,b show the ultrasound measurement scenarios of diaphragmatic movement. After deep anesthesia, the pig’s breathing essentially ceased. Correspondingly, the displacement of the right and left diaphragms was 0 cm (Figure 5c,d). The designed DP system sends the same stimulation parameters (6 V, 50 Hz, 150 μs, 0.5 s, 1.5 s) to the phrenic nerves on both sides, which can increase the bilateral diaphragmatic displacement from 0 cm to 1.3 cm (Figure 5e) and 1.1 cm (Figure 5f), respectively, with each breath.

**Figure 6:**
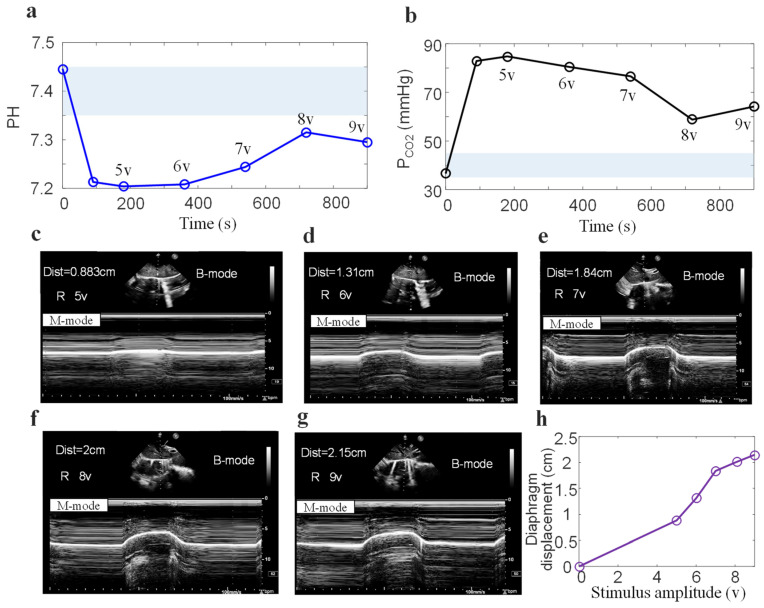
Effects of different stimulus amplitudes on blood gas and diaphragmatic displacement. (**a**) pH values and (**b**) P_CO2_ from discrete arterial blood gas, Diaphragmatic displacement visible via M-mode ultrasound at stimulation amplitudes of (**c**) 5 V, (**d**) 6 V, (**e**) 7 V, (**f**) 8 V, and (**g**) 9 V per breath. (**h**) Diaphragmatic displacement measured by M-mode ultrasound of the same subject at different stimulation amplitudes.

Figure 6 shows changes in arterial blood gas (pH, P_CO2_) and diaphragmatic ultrasound displacement at different stimulation amplitudes (5–9 V). The light blue shadow represents the standard range of normal values for arterial blood gas index (Figure 6a,b). During mild anesthesia, pH (7.446) and P_CO2_ (36.6 mmHg) are both within the normal range. Deep anesthesia resulted in respiratory depression in the pigs, causing blood gas parameters to be outside the normal range. Respiratory acidosis is directly manifested by an increase in P_CO2_. Increasing the stimulation amplitude and duration improved blood gas parameters. At 12 min, the partial pressure of P_CO2_ decreased significantly, while at 15 min, there was a slight increase. Figure 6c,d illustrate the ultrasound diaphragm displacement under different stimulation amplitudes (5–9 V). The increase in diaphragmatic displacement was greater when the stimulus amplitude was between 5 V and 7 V. When the stimulus amplitude was greater than 7 V, the increase in diaphragmatic displacement decreased significantly.

**Figure 7:**
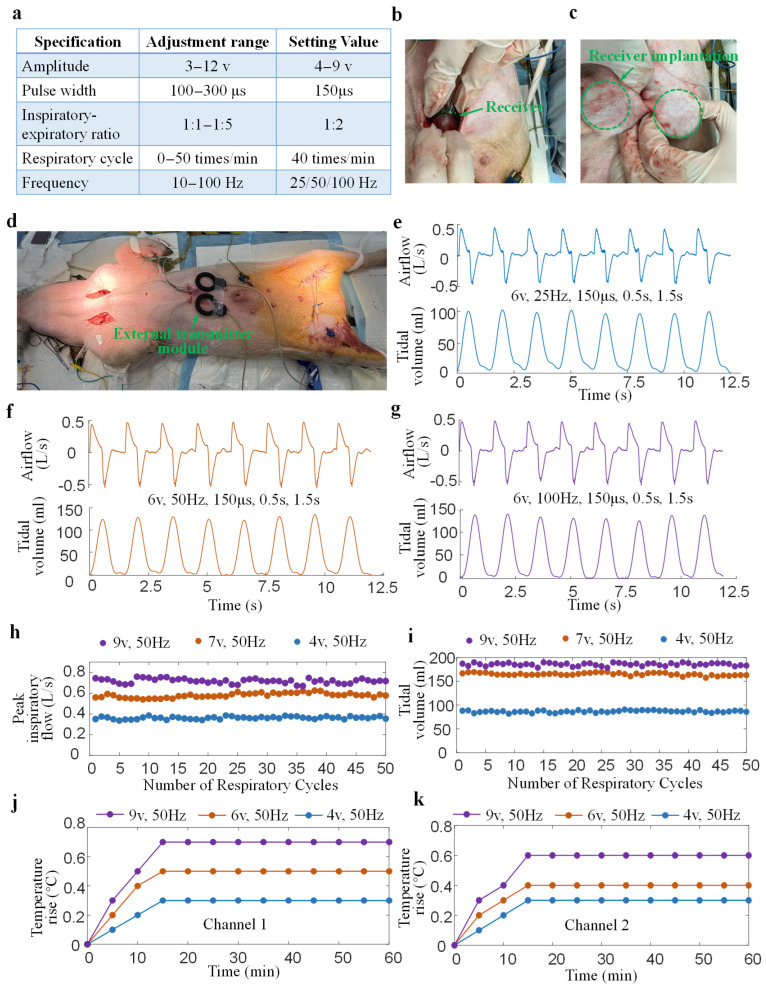
The proposed diaphragmatic pacemaker’s ability to maintain respiratory ventilation. (**a**) Parameter settings in diaphragmatic pacing experiments. (**b**,**c**) Subcutaneous implantation of the internal receiver in the abdomen. (**d**) The scene during the experiment where the external transmitter was placed above the receiver implanted under the skin. (**e**) Respiratory airflow and corresponding tidal volume under a certain stimulus parameter (6 V, 25 Hz, 150 μs, 0.5 s, 1.5 s). (**f**,**g**) Respiratory airflow and corresponding tidal volume at two different stimulation frequencies (50 Hz and 100 Hz). (**h**,**i**) Peak airflow and tidal volume maintenance over 50 pacing cycles at three different stimulation amplitudes (4 V, 7 V, 9 V). (**j**,**k**) Tissue time–temperature rise test after implantation of the two implanted receivers.

Figure 7 illustrates the ability of the designed DP system to maintain respiratory ventilation during animal experiments. Figure 7a shows the range of adjustable stimulation parameters for the DP system and the stimulation parameters set in animal experiments. Multiple stimulus amplitudes (4 V, 6 V, 7 V, 9 V) and frequencies (25 Hz, 50 Hz, 100 Hz) were selected to influence respiratory airflow and tissue temperature rise. The remaining stimulation parameters, including pulse width (150 μs), inspiratory-expiratory ratio (1:2), and respiratory cycle (1.5 s), remained constant. Figure 7b,c show the scene of two receivers being implanted under the skin of a pig’s abdomen. Figure 7d shows the test scenario of a diaphragmatic pacing experiment in which two external controllers are placed directly above two receivers that have been implanted under the skin of a pig. Figure 7e–g show the airflow and tidal volume waveforms of eight consecutive respiratory cycles induced by different stimulation frequencies (25 Hz, 50 Hz, 100 Hz) and with the other stimulation parameters being the same (6 V, 150 μs, 0.5 s, 1.5 s). It can be observed that all airflow peaks remain essentially consistent, without significant increases or decreases. Low stimulation frequency causes incomplete contraction of the diaphragm, resulting in spikes in the respiratory airflow waveform (Figure 7e). Increasing the stimulation frequency smooths the respiratory airflow (Figure 7f,g). Intuitively, for the same stimulus amplitude, the peak airflow induced by different frequencies remains basically the same. However, different stimulus frequencies significantly alter the tidal volume. The average tidal volumes at 25 Hz, 50 Hz, and 100 Hz are 109.22 mL, 126.93 mL, and 131.18 mL, respectively. Figure 7h,i show the changes in peak airflow and tidal volume over 50 consecutive respiratory cycles under three diaphragmatic pacing stimulation parameters. It can be observed that increasing the stimulation parameters significantly increases the peak airflow and tidal volume. The peak airflow and tidal volume remained essentially unchanged over 50 consecutive cycles at three different stimulus amplitudes (4 V, 7 V, 9 V). This demonstrates that the designed DP system has a good ability to maintain respiratory ventilation. Figure 7j,k show the temperature rise changes when the two receivers were implanted in the abdomen of a pig. The temperature change was mainly concentrated in the first 20 min of the experiment and remained basically constant after 20 min. An increase in the amplitude of the stimulus leads to an increase in temperature rise. The maximum temperature rise was 0.7 °C at 60 min after implantation, which is lower than the 2 °C limit for tissue temperature rise specified by the ISO standard.

**Figure 8:**
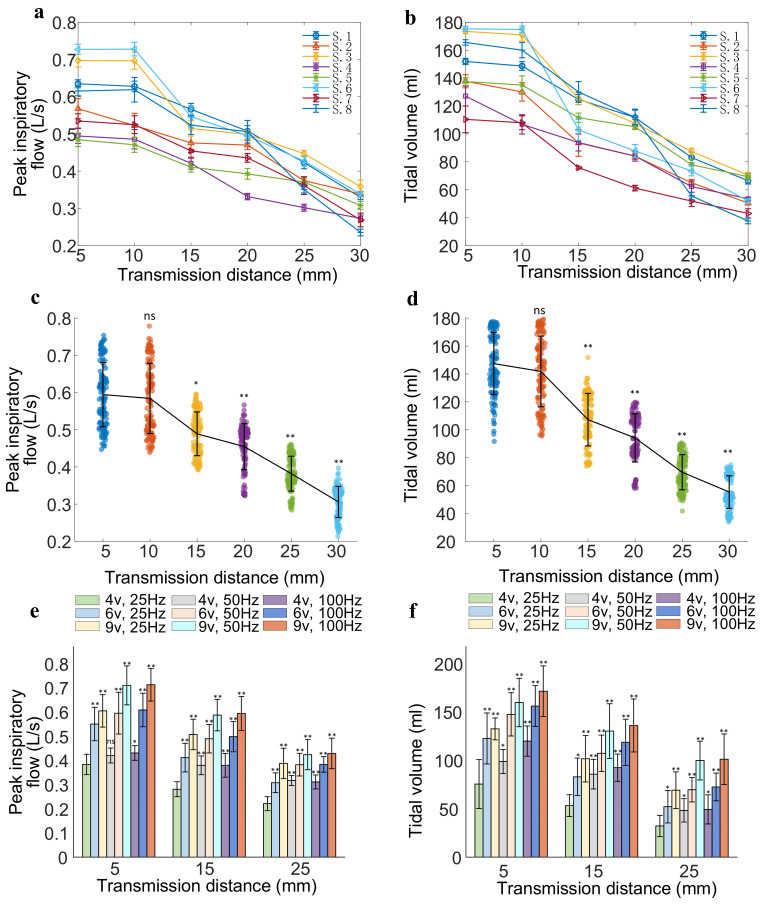
Peak airflow and tidal volume caused by the DP system at different transmission distances and stimulation parameters. (**a**,**b**) Changes in peak airflow and tidal volume at various transmission distances during diaphragmatic pacing in each pig under the same stimulus parameters (6 V, 150 μs, 50 Hz, 0.5 s, 1.5 s). (**c**,**d**) Trends in peak airflow and tidal volume changes at various transmission distances, with the same stimulus parameters (6 V, 150 μs, 50 Hz, 0.5 s, 1.5 s) during DP in all experimental subjects. (**e**,**f**) Comparison of peak airflow and tidal volume under different stimulus amplitudes (4 V, 6 V, 9 V), frequencies (25 Hz, 50 Hz, 100 Hz), and transmission distances (5 mm, 15 mm, 25 mm). n = 8; ns: not significant; * *p* < 0.05, ** *p* < 0.01.

Figure 8 shows the changes in peak airflow and tidal volume of eight anesthetized pigs at different transmission distances and stimulation parameters. Figure 8a,b show the variation in mean and standard deviation of peak respiratory flow and tidal volume for each pig with 15 consecutive respiratory cycles at various transmission distances (5–30 mm). Figure 8c,d are a compilation of data from all experimental subjects. Under the same stimulus parameters, increasing the transmission distance decreases peak airflow and tidal volume. When the transmission distance exceeds 15 mm, peak airflow and tidal volume decrease more significantly (*p* < 0.05). With the stimulation parameters kept constant (6 V, 150 μs, 50 Hz, 0.5 s, 1.5 s), the peak airflow and tidal volume for all subjects at a transmission distance of 5 mm were 0.594 ± 0.086 L/s (95% CI 0.534–0.654) and 147.39 ± 22.08 mL (95% CI 128.93–165.85), respectively. However, at a transmission distance of 30 mm, the peak airflow and tidal volume for all subjects decreased to 0.306 ± 0.041 L/s (95% CI 0.272–0.340) and 55.35 ± 11.68 mL (95% CI 45.59–65.11), respectively. Figure 8e,f show a comparison of peak airflow and tidal volume at different transmission distances (5 mm, 15 mm, 25 mm), stimulus amplitudes (4 V, 6 V, 9 V), and frequencies (25 Hz, 50 Hz, 100 Hz). It can be observed that when the stimulation frequency and transmission distance remain unchanged, the stimulation amplitude significantly increases the peak airflow and tidal volume (*p* < 0.05). While keeping the stimulation amplitude (4 V) and transmission distance (15 mm) constant, increasing the stimulation frequency increases peak airflow and tidal volume (*p* < 0.05).

**Figure 9:**
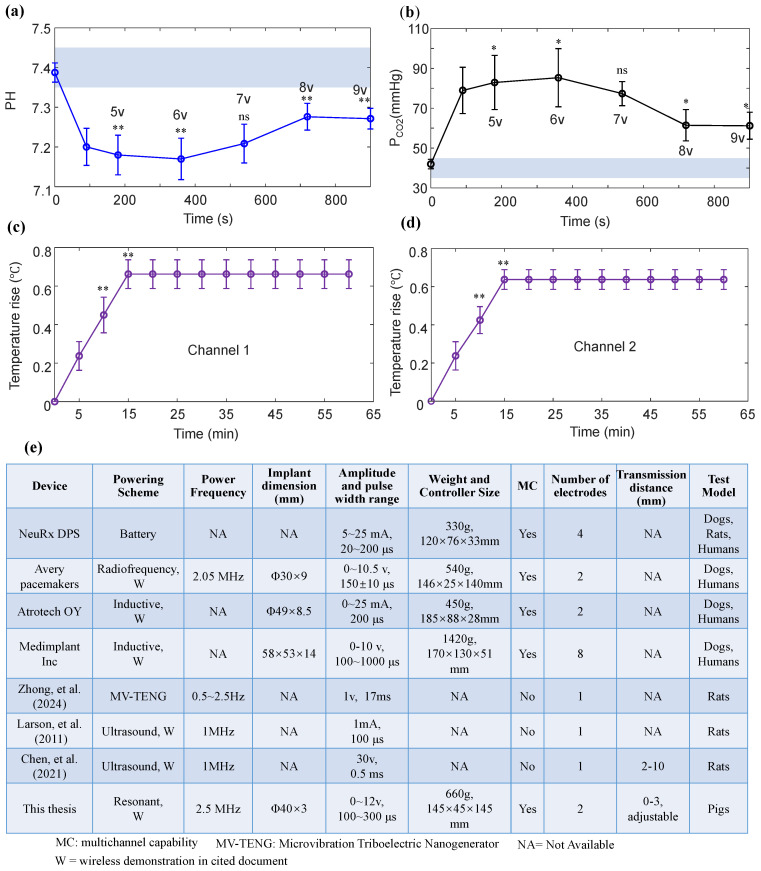
Blood gas and temperature rise tests and comparisons with other devices. (**a**) pH values and (**b**) P_CO2_ from the discrete arterial blood gas of eight pigs. (**c**,**d**) Temperature changes at different times after two receivers were implanted in eight pigs (stimulus amplitude: 9 V). (**e**) Comparisons with existing diaphragm pacemakers and wireless implantable neurostimulation systems [20,36,37,38,39,40]. n = 8; ns: not significant; * *p*< 0.05, ** *p* < 0.01.

Figure 9 shows the blood gas temperature rise tests of all subjects and comparisons with other devices. Figure 9a,b illustrate the changes in pH and P_CO2_ in eight pigs under different stimulus amplitudes. Increasing the anesthetic concentration resulted in a decrease in pH (7.20 ± 0.047, 95% CI 7.167–7.232) and an increase in P_CO2_ (78.96 ± 11.63 mmHg, 95% CI 70.901–87.019). Compared with the respiratory depression state, diaphragmatic pacing (stimulus parameters: 8 V/9 V, 150 μs, 50 Hz, 0.5 s, 1.5 s) significantly increased arterial pH, while markedly reducing P_CO2_ (*p* < 0.05).

Figure 9c,d show the temperature rise changes of all subjects after two receivers were implanted. Compared with 5 min after implantation, the temperature rise of channel 1 was significantly increased at 10 min (0.45 ± 0.09 °C, 95% CI 0.388–0.512) and 15 min (0.66 ± 0.07 °C, 95% CI 0.611–0.708) (*p* < 0.05). Twenty minutes after the two receivers were implanted, there was no significant change in temperature rise. The maximum temperature rise of the two receivers did not exceed 2 °C.

Figure 9e is a comparison table of the DP system proposed in this paper with existing diaphragm pacemakers and wireless implantable neurostimulation systems. The DP system proposed in this paper essentially performs the functions of existing commercial diaphragm pacemakers [36,37,38], such as Avery pacemakers, Atrotech OY, and Medimplant Inc. A novel power supply method (MV-TENG) enables diaphragmatic pacing [20]. However, its stimulation parameters, such as frequency for 0.5~2.5 Hz, amplitude for 1 V, and pulse width for 17 ms, do not meet the requirements for clinical use. Neurostimulators powered by wireless ultrasound transmission [39,40] cannot achieve bilateral diaphragmatic pacing when the number of channels is 1. Furthermore, it has not been effectively validated in large-animal models. The stimulation parameters and the number of stimulation channels in the DP system proposed in this paper are basically consistent with those of commercially available diaphragmatic pacemakers. Resonant wireless power transfer offers superior transmission stability compared to inductive power transfer. While the DP system proposed in this paper is not optimal in terms of size and weight compared to commercial products, it is not the worst either. For example, regarding the size of the implanted receiver, the implant dimensions in this paper are significantly smaller than those of Atrotech OY.

## 4. Discussion

This study independently developed an implantable diaphragmatic pacemaker that integrates electromagnetic resonant-coupling technology with a wireless-charging system for diaphragmatic nerve modulation. The use of electromagnetic coupling technology enables non-contact energy transmission, with advantages such as high energy transmission efficiency, a moderate penetration depth, and minimal electromagnetic interference with surrounding tissues [41,42]. Compared to inductive-coupling wireless charging, the electromagnetic resonant-coupling system has a longer transmission distance. It is better suited to the different positions of the implantation site and the transmitter on the body surface in clinical applications [41,42,43].

We reviewed the existing literature and user manuals on commercial diaphragm pacemakers but found no technical parameters for wireless power transfer efficiency. Therefore, there is no clear reference point for wireless transmission efficiency in existing wireless DP systems. In wireless power transmission for small implantable medical devices, the transmission efficiency is typically 10–50% due to limitations in coil size and the fact that the system power required is usually in the mW range [44,45,46]. The wireless power transmission efficiency of the implantable diaphragm pacemaker designed in this paper reached approximately 40% at a transmission distance of 15 mm. Therefore, the transmission coil selected in this paper meets the available parameters for existing implantable medical devices.

Temperature safety is a key prerequisite for the clinical translation of implantable electronic devices. This study used finite-element analysis to calculate the thermal effect on tissue temperature of the receiver under working conditions within the implanted body. The transmission power of implantable neurostimulators is mostly in the range of tens to hundreds of milliwatts [47,48]. Therefore, it is reasonable to set the emission power range of 0.5–1.5 W when simulating the tissue temperature. In continuous 1-h power output mode, the maximum temperature of the surrounding tissue of the receiver does not exceed 2 °C under various implantation conditions. The temperature rise range meets the temperature safety requirements for implantable medical devices specified in ISO 14708-1 of the International Organization for Standardization [31]. We also found that, under the same input power and transmission distance, the temperature rise increased by 0.848–1.062 °C when the relative position of the coil group was offset compared to the aligned case. This pattern of change is consistent with the previous study [49], demonstrating the rationality of the simulation conditions set in this study. The specific absorption rate (SAR) of human tissue during wireless power transmission has also been proven to be within a safe threshold [19,49]. The simulation results of the temperature rise range and SAR value verified the safety of the DP system. It provided a theoretical basis for the implantability of the device.

In vitro functional testing used pork tissue as a simulation medium because its water content, density, and dielectric properties are similar to those of human soft tissue. This effectively simulates the energy transmission and stimulus response environment after the receiver is implanted in the body [25,50]. Test results show that the device can stably deliver stimulation pulses over a range of transmission distances (5–35 mm). Previous studies have shown that the intensity of in vivo neural electrical stimulation is usually in the mA range [51,52]. The output current amplitude (1.8–2.6 mA) of the two receivers in the designed DP system is sufficient for some neural electrical stimulation experiments [51,52,53,54]. This test provides a reliable basis for subsequent in vivo experiments on anesthetized pigs.

The diaphragm anatomy, nerve innervation, and respiratory physiology of pigs are similar to those of humans [55,56]. In vivo experiments using eight deeply anesthetized pigs provide an ideal animal model for evaluating the performance of the proposed DP system. The advantages of the Cuff electrode [57], such as its flexibility in adapting to nerve size and its excellent biocompatibility, provide key support for achieving precise, efficient, and safe phrenic nerve stimulation. The experiment collected respiratory airflow response and diaphragmatic ultrasound displacement data under different stimulation parameters. Under certain stimulation parameters (6 V, 50 Hz), the tidal volume generated by diaphragmatic pacing can reach 6.35 mL/kg (Figure 7f), which is comparable to the tidal volume level (6–8 mL/kg) of mechanical ventilation on a ventilator [58]. Meanwhile, the diaphragm displacement measured by ultrasound was 0.883–2.15 cm (Figure 6). Other studies have also reported diaphragmatic excursion of 1.73 ± 0.17 cm and 1.99 ± 0.46 cm during DP in pigs, with baseline measurements of 0.87 ± 0.13 cm and 0.09 ± 0.04 cm before DP [59,60].

This confirms that the diaphragmatic contraction caused by the DP system is effective. More importantly, blood gas analysis results confirmed that increasing the stimulation amplitude effectively increases pH and reduces PaCO_2_ in arterial blood. These results confirm that the implanted diaphragmatic pacemaker can effectively activate diaphragmatic contraction and generate respiratory movements that meet physiological needs. This provides a new technical solution for the clinical treatment of respiratory muscle paralysis, such as high spinal cord injury and amyotrophic lateral sclerosis.

This study employed an acute porcine model of respiratory arrest induced by deep anesthesia, primarily to validate the acute ventilation function and parameter response patterns of diaphragmatic pacing under conditions of central driver deficiency. Eliminating interference from spontaneous respiration provided a stable and reliable experimental basis for optimizing pacing parameters. Chronic pathological models are more suitable for research on long-term adaptation, diaphragmatic remodeling, and neural plasticity. These two animal models represent a progressive research approach, rather than being mutually exclusive. The deep anesthesia respiratory depression model provides the necessary preliminary experimental basis for the eventual application of diaphragmatic pacing in patients with chronic respiratory dysfunction.

This study has some limitations. A control group with commercial diaphragmatic pacemakers and data from chronic implantation experiments are needed to further evaluate its effectiveness. It is a short-term acute experiment (observation duration < 48 h). The validation of diaphragm pacing stimulation parameters is based on a healthy pig model, without considering pathological conditions such as diaphragm atrophy, muscle fiber type conversion, or decreased nerve sensitivity in experimental subjects with chronic respiratory failure. Future research should focus on further optimization in surgical techniques, safety evaluation after long-term implantation, and device performance. The phrenic nerve is located in the neck and chest, and surgical exposure and electrode implantation require extremely high precision and skill. Efforts should be made to promote minimally invasive surgery to reduce surgical trauma. Monitoring for issues such as infection and equipment malfunction remains a challenge for long-term safety management. Before clinical trials, the sample size should be expanded to conduct long-term implantation experiments (>3 months), monitoring histocompatibility, device failure rate, and respiratory function recovery. 

Furthermore, the limited application of implantable diaphragm pacing (IDP) stems from narrow indications, reliance on external RF power, unstable electrode-neuron interface, and high surgical trauma. We proposed the following solutions: (1) expanding to multi-target nerve-muscle stimulation to cover peripheral respiratory muscle disorders; (2) position the implanted antenna adjacent to the sternum (a bone) to maximize power dissipation in a human scenario; (3) employing nanomodified flexible electrodes and minimally invasive implantation to reduce complications; (4) establishing closed-loop adaptive stimulation and remote monitoring to improve reliability and quality of life. Through technological innovation and clinical strategy optimization, IDP can be extended from a few central respiratory failure cases to broader scenarios, such as respiratory muscle rehabilitation and weaning assistance, in the future.

## 5. Conclusions

This study successfully developed an implantable diaphragmatic pacemaker (DP) based on electromagnetic resonant-coupling and wireless-charging technology. The feasibility and efficacy of the device were fully validated through tissue-temperature simulations, in vitro functional tests, and in vivo experiments in eight anesthetized pigs. This innovative and safe DP system provides a novel technical solution for replacing or assisting mechanical ventilation, thereby improving the prognosis of patients with respiratory dysfunction. Moving forward, further optimization of device design and advancement of translational clinical research are expected to drive significant breakthroughs in respiratory support therapy.

## Data Availability

The original contributions presented in this study are included in the article. Further inquiries can be directed to the corresponding authors.
